# Modulation of E-cadherin expression promotes migration ability of esophageal cancer cells

**DOI:** 10.1038/srep21713

**Published:** 2016-02-22

**Authors:** Shujun Li, Xuebo Qin, Song Chai, Changbao Qu, Xiaolu Wang, Helin Zhang

**Affiliations:** 1 Department of Thoracic Surgery, The Second Hospital of Hebei Medical University, Shijiazhuang, 050051, China; 2 Department of Thoracic Surgery, Hebei Chest Hospital, Shijiazhuang, 050051, China; 3 Cancer Center, The Second Hospital of Hebei Medical University, Shijiazhuang, 050051, China

## Abstract

Losing the E-cadherin plays an important role in the metastasis of cancer. The regulation of the expression of E-cadherin is unclear. Circadian rhythm alteration is associated with the pathogenesis of a number of cancers. This study aims to investigate the role of one of the circadian proteins, period-2 (Per2) in repressing the expression of E-cadherin in esophageal cancer (esophageal cancer). We observed that the levels of circadian protein Per2 were significantly increased and E-cadherin was significantly decreased in the tissue of human esophageal cancer with metastasis as compared with non-metastatic esophageal cancer. Overexpression of Per2 in the esophageal cancer cells markedly repressed the expression of E-cadherin. The pHDAC1 was detected in human esophageal cancer with metastasis, which was much less in the esophageal cancer tissue without metastasis. Overexpression of Per2 increased the levels of pHDAC1 as well as the E-cadherin repressors at the E-cadherin promoter locus. Overexpression of Per2 markedly increased the migratory capacity of esophageal cancer cells, which was abolished by the inhibition of HDAC1. We conclude that Per-2 plays an important role in the esophageal cancer cell metastasis, which may be a novel therapeutic target for the treatment of esophageal cancer.

Esophageal cancer is one of the leading causes in human death. The therapeutic effect of esophageal cancer is largely related to the pathological stages at diagnosis^1^. Because of the anatomical feature, many esophageal cancer cases are in the advanced stages with metastasis at diagnosis^2^. The underlying mechanism of cancer metastasis is to be further investigated. Despite the research in esophageal cancer advanced rapidly in last a few decades, the therapeutic effect on this cancer is still poor. The long term survival rate of esophageal cancer patients is dismay currently; the five-year survival rate is less than 20%^3^,^4^. Therefore, it is necessary to understand the biological feature of esophageal cancer to predict clinical behavior and identify novel molecular targets for therapy.

Cancer metastasis is the spread of a cancer from one organ to another not directly connected with it. Three kinds of motion are involved in cancer metastasis, including collective motility, mesenchymal-type movement, and amoeboid movement^5^. E-cadherin (E-cadherin) is associated with the epithelial-mesenchymal transition of cancer. Cadherins are a class of type-1 transmembrane proteins. E-cadherin is epithelial origin. Loss of E-cadherin function or expression has been implicated in cancer progression and metastasis^6^. E-cadherin downregulation decreases the strength of cellular adhesion within a tissue, resulting in an increase in cellular motility. However, the causative factors down regulating E-cadherin need to be further elucidated.

It is reported that Period 2 protein (Per2) and E-cadherin mRNA levels show robust circadian oscillation^7^. The fact implicates that the circadian clock alteration may be involved in regulating the expression of E-cadherin. It is proposed that circadian rhythm disruption is associated with cancer; such as Okabe *et al* indicate that HIF1α enhances the amplitude of the Per2 circadian rhythm in renal cancer cell lines^8^,^9^. Therefore, we hypothesize that the circadian proteins may modulate the expression of E-cadherin in esophageal cancer cells to promote the esophageal cancer cell migratory capacity. Thus, we carried out the present study. The results showed that high levels of Per2 were detected in the surgically removed esophageal cancer tissue. Overexpression of Per2 in esophageal cancer cells suppressed the expression of E-cadherin and promoted the migratory capacity of esophageal cancer cells.

## Results

### Expression of Per2 and E-cadherin was detected in esophageal cancer with metastasis

**Figure 1:**
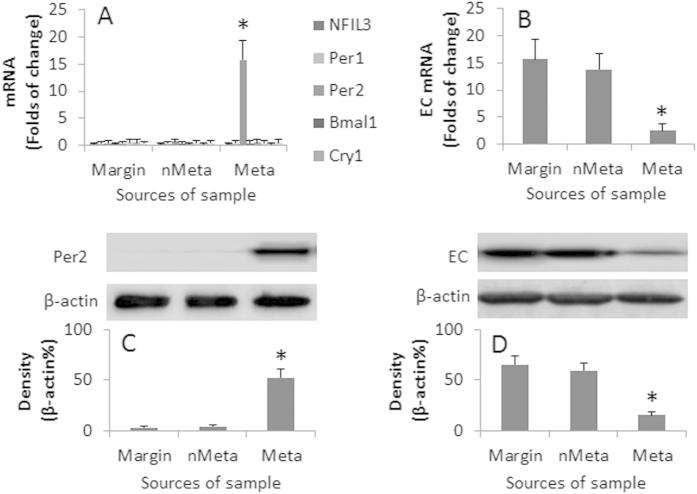
Expression of Per2 and EC in Eca cells. Eca cells were isolated from the surgically removed Eca tissue of 20 Eca patients. The RNA and proteins were extracted from the marginal tissue (Margin), Eca cells from Eca without metastasis (nMeta), and Eca cells from Eca with metastasis (Meta) were analyzed by RT-qPCR and Western blotting. Each sample contained 1 × 10^5^ cells. (**A**) the bars indicate the mRNA levels of circadian molecules. (**B**) the bars indicate the mRNA levels of EC. (**C**) the Western blots indicate the protein levels of Per2. (**D**) the Western blots indicate the protein levels of EC. The bars below the Western blots indicate the integrated density of the blots. Samples from individual patients were analyzed separately. The data are representatives of the results from 20 samples. The data of bars are presented as mean ± SD. *p < 0.01, compared to the marginal group. (The full length gel graphs are presented in the relevant information).

The circadian clock disruption is associated with the pathogenesis of cancer^10^. We wondered if the circadian clock disruption was associated with esophageal cancer metastasis. To this end, we collected surgically removed esophageal cancer tissue from 20 esophageal cancer patients. The esophageal cancer cells were negatively isolated by MACS and subjected to RT-qPCR to detect the expression of circadian clock molecule mRNA, including NFIL3, Per1, Per2, Bmal1, Cry1, Cry2, Clock and Npas2. The results showed that the expression of Per2 was uniquely increased in the esophageal cancer with metastasis, but not in the esophageal cancer without metastasis, nor in the marginal tissue (Fig. 1A). Since a decrease in E-cadherin is an important factor in the pathogenesis of cancer metastasis^11^, we also assessed the expression of E-cadherin in the esophageal cancer cells and the marginal tissue. The results showed that the expression of E-cadherin was markedly less in esophageal cancer with metastasis that of the esophageal cancer without metastasis and the marginal tissue (Fig. 1B). The results were confirmed by the data of Western blotting (Fig. 1C,D).

### Per2 represses the expression of E-cadherin in esophageal cancer cells

**Figure 2:**
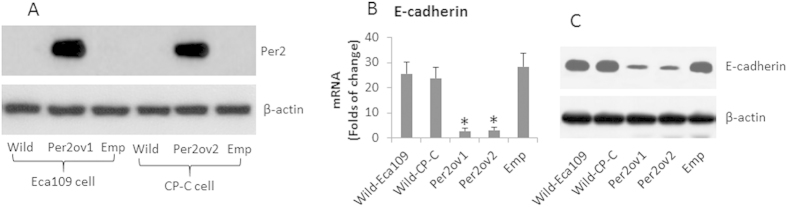
Overexpression of Per2 suppresses EC expression in Eca cells. Eca109 cells were transfected with Per2 plasmids or empty plasmids. The cell extracts were then analyzed by RT-qPCR and Western blotting. (**A**) the Western blots indicate the overexpression of Per2 in Eca109 and CP-C cells. (**B**) the bars indicate the mRNA levels of EC (mean ± SD; *P < 0.01, compared with the wild group). (**C**) the Western blots indicate the protein levels of EC. Wild: Wild Eca109 cells. Per2ov: Eca109 cells with Per2 overexpression in Eca109 (Per2ov1) and CP-C (Per2ov2) cells. Emp: Eca109 cells were transfected with empty plasmids using as a control. The data are representative of 3 independent experiments. (The full length gel graphs are presented in the relevant information).

The results of Fig. 1 suggest that Per2 represses the expression of E-cadherin in esophageal cancer cells. To test the inference, we overexpressed the Per2 in Eca109 cells and CP-C cells (Two esophageal cancer cell lines) (Fig. 2A), which markedly repressed the expression of E-cadherin in the esophageal cancer cells (Fig. 2B,C).

### Per2 overexpression enhances pHDAC1 in esophageal cancer cells

**Figure 3:**
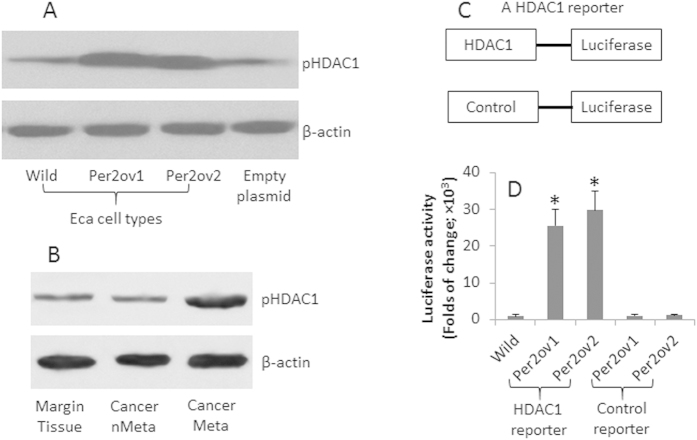
Per2 increases HDAC1 in esophageal cancer cells. Eca109 and CP-C cells were overexpressed with Per2; the cell extracts were analyzed by Western blotting to assess the levels of pHDAC1. (**A**) the Western blots indicate the levels of pHDAC1. (**B**) the immune blots indicate the levels of pHDAC1 in the samples of human esophageal cancer. (**C**) the sketch show constructs of a HDAC1 reporter and a control reporter. D, the Per2-overexpressing Eca cells were transfected with the HDAC1 reporters and analyzed by luciferase activity assay. The bars indicate the luciferase activity (mean ± SD; *p < 0.01, compared with the wild group). The data are representative of 3 independent experiments. (The full length gel graphs are presented in the relevant information).

HDAC1 is a histone deacetylase; it plays a role in cancer growth^12^ and is involved in the regulation of a large number of gene transcription. It is suggested that HDAC1 is involved in the regulation of circadian clock event^13^. We wondered if HDAC1 was involved in the Per2-repressed E-cadherin expression in esophageal cancer cells. To this end, we assessed the levels of pHDAC1 in esophageal cancer cells with or without Per2 overexpression. The results showed that higher levels of pHDAC1 were detectable in wild esophageal cancer cells, which was markedly increased in the Per2-overexpressing esophageal cancer cells (Fig. 3A). To enforce the results, we collected samples from patients with esophageal cancer. The extracts were analyzed by Western blotting. The results showed that the pHDAC1 was detectable in the marginal tissue and esophageal cancer tissue without metastasis, which was significantly increased in the esophageal cancer tissue with metastasis (Fig. 3B). The results implicate that Per2 may increase the levels of pHDAC1. To test the inference, we constructed a reporter gene of HDAC1. The Eca cells of Eca109 and CP-C were transfected with the HDAC1 reporters or control reporters. As shown by luciferase assay, the overexpression of Per2 markedly increased the HDAC1 expression in the Eca cells.

### HDAC1 suppresses E-cadherin transcription in esophageal cancer cells

**Figure 4:**
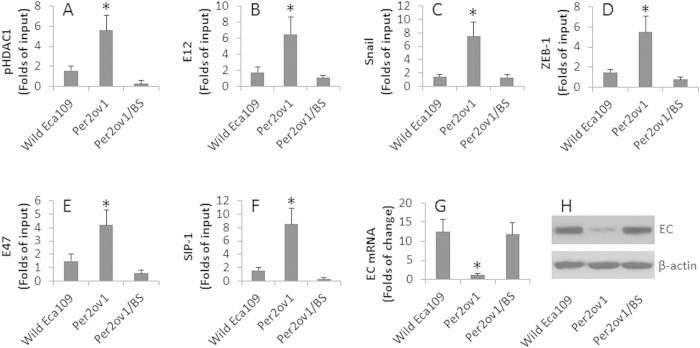
Per2 modulates EC transcription. Eca109 cells were overexpressed Per2 (Per2ov1) with or without treating with butyrate sodium (BS, an inhibitor of HDAC1; 1 mg/ml). The cell extracts were analyzed by ChIP, RT-qPCR and Western blotting. (**A–F**) the bars indicate the levels of pHDAC1 and EC transcription repressors at the EC promoter locus. (**G**) the bars indicate the levels of EC mRNA in Eca109 cells. H, the immune blots indicate the levels of EC protein in Eca109 cells. The data of bars are presented as mean ± SD. *p < 0.01, compared with the wild group. The data are representative of 3 independent experiments. (The full length gel graphs are presented in the relevant information).

We next took a further insight into the mechanism by which Per2 modulating the gene transcription of E-cadherin in esophageal cancer cells. We firstly overexpressed Per2 in Eca109 cells, the cell extracts of the cells were analyzed by ChIP. The results showed that high levels of pHDAC1 were detected at the E-cadherin promoter locus, which were abolished in the presence of BS (Fig. 4A). Several E-cadherin repressors have been characterized. These repressors play an important role in the suppression of E-cadherin^14^,^15^,^16^,^17^. We wondered if the overexpression of Per2 promoted a role in the up regulation of the E-cadherin repressors in esophageal cancer cells. To this end, we assessed the E-cadherin repressors in the Eca109 cells with or without Per2 overexpression. As shown by ChIP assay, the E-cadherin repressors, including E12, Snail, ZEB-1, E47, SIP-1, were detectable at the E-cadherin promoter locus of wild Eca109 cells, which were markedly enhanced in Eca109 cells with the Per2 overexpression, which were abolished in the presence of BS in the culture (Fig. 4B–F). Further analysis showed that the overexpression of Per2 inhibited the expression of E-cadherin in Eca109 cells, which could be prevented by the presence of BS in the culture (Fig. 4G,H).

### Per2 facilitates migration of esophageal cancer cells

**Figure 5:**
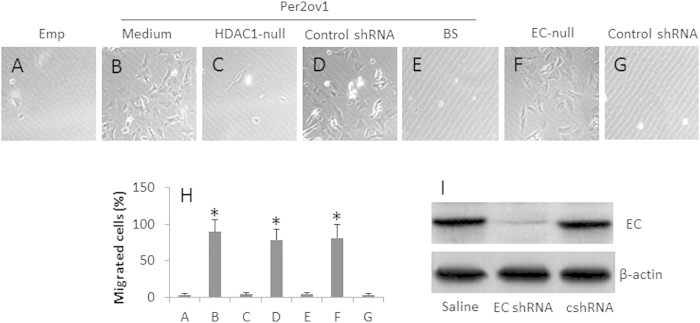
Per2 promotes Eca cell migration. Eca109 cells with Per2 overexpression (Per2ov1), or transfected with empty plasmids (Emp) were cultured in Transwells. (**A–G**) the representative images (×200) show the migrated cells. (**H**) the bars indicate the summarized total migrated cells in (**A–G**) (mean ± SD; *p < 0.01, compared the group transfected with empty plasmids). (**I**) the Western blots show the EC gene knockdown results. The data are representative of 3 independent experiments. (The full length gel graphs are presented in the relevant information).

We next observed the biological aspect of Per2 in esophageal cancer cells. It is suggested that the decreases in E-cadherin facilitates cancer metastasis^11^,^18^. The above results indicate the overexpression of Per2 decreases the expression of E-cadherin in esophageal cancer cells, which implicate these Per2^+^ esophageal cancer cells might have the high migration property. To test the inference, we cultured esophageal cancer cells with or without the overexpression of Per2 in a Transwell system. The results showed that the overexpression of Per2 markedly increased the migration of esophageal cancer cells, which was abolished by transfected with empty plasmids, or knockdown of the HDAC1 gene, of knockdown the E-cadherin gene, respectively (Fig. 5). The results suggest that overexpression of Per2 facilitates the esophageal cancer cell invasion.

## Discussion

Metastasis is a critical checkpoint in the prognosis of cancer^19^. Thus, to understand the mechanism by which cancer cells migrate to remote organs or tissue is of significance in the prevention of metastasis of cancer. The present data show that one of the circadian proteins, Per2, is highly expressed in esophageal cancer tissue, which is highly correlated with the metastasis of esophageal cancer and the suppression of the expression of E-cadherin in esophageal cancer cells. The alteration of circadian rhythm frequently occurs in human daily life, such as jet lag and day-night-shift. Perturbation of circadian rhythm may alter the expression of circadian protein, and disturb physiological functions^20^. Published data indicate that the circadian rhythm disruption is associated with numberous aspects of health and to induce pathological conditions^21^, such as metabolisc diseases, cardiovascular diseases, thrombosis^22^, or the pathogenesis of cancer^9^,^23^. The present data show that the levels of Per2 are markedly high in esophageal cancer with metastasis, implying such an unusual expression of Per2 may be associated with esophageal cancer metastasis; the underlying mechanism needs to be further investigated.

The alternation of Per2 expression during the perturbation of circadian rhythm has been documented. Dalvin *et al* reported that perturbation increased the expression of Per2 in the iris-ciliary body complex in a circadian pattern^24^. More *et al* indicated that, after stimulated with the glucocorticoid receptor antagonist RU486, jejunal explants from mice displayed 1.5– to 2-fold circadian oscillations of Per2 protein abundance that persisted for up to ~84 hours^25^. Uisman *et al* indicate that hepatic metastases of C26 colon carcinoma with a disrupted circadian rhythm phase shift liver and kidney tissue clocks, suggesting the circadian timing system plays an essential role in the development of cancer^26^.

Yamato *et al* reported that E-cadherin protein oscillated circadianly under ad libitum feeding; it was altered in response to the restricted feeding^7^. Our data show a novel aspect of Per2 expression in esophageal cancer that high levels of Per2 were detected in the cancer tissue of metastasis. We also found that the E-cadherin expression was markedly lower in esophageal cancer with metastasis. E-cadherin is one of the proteins between epithelial cells, by which the epithelial cells connect each other. Cancer cells from epithelial cells still keep such a histological feature. Loss of cell-cell connection protein in cancer tissue is well documented in a number of cancers^27^. Liu *et al* also noted that E-cadherin status was significantly associated with esophageal cancer invasion, metastasis and prognosis^28^. Our data are in line with Liu’s report by showing a close relation between lower expression of E-cadherin and esophageal cancer metastasis.

One of the novel points of the present data is that Per2 expression is negatively associated with the decrease in E-cadherin expression in esophageal cancer cells. Per2 is one of the circadian proteins; its expression is regulated by the circadian oscillator. The roles of clock genes in the maintenance of body tissues have been recognized and a potential link between the circadian rhythm disruption and cancer has been proposed^29^. The present data provide further evidence that overexpression of Per2 promotes the migratory capacity of esophageal cancer cells. Others also propose that Per2 may play important roles in tumor development, invasion and prognosis, and Per2 may serve as a novel prognostic biomarker of human gastric cancer^30^.

HDAC1 is a histone deacetylase. The present data show that overexpression of Per2 increased the pHDAC1, indicating that Per2 is associated with the HDAC1 expression. The data show that HDAC1 played a key role in repressing the E-cadherin gene transcription via increasing the E-cadherin repressors. The E-cadherin repressors play important rle in suppression of E-cadherin^14^,^15^,^16^,^17^. Meng *et al* also show that HDAC1 promotes the tumorigenicity of the ovarian cancer^31^. In line with our data, Kou *et al* indicate that HDAC1 promotes cancer cell migration and invasion^32^. The information implies that the inhibition of HDAC1 may suppress cancer metastasis. The inference is supported by our further experimental data; the presence of sodium butyrate (a HDAC1 inhibitor) markedly inhibits the migratory capacity of esophageal cancer cells.

In summary, the present data show that human esophageal cancer cells with metastasis express high levels of Per2; Per2 suppresses the expression of E-cadherin in esophageal cancer cells. HDAC1 is involved in the inhibitory process of E-cadherin induced by Per2; blocking HDAC1 inhibits the migratory capacity of esophageal cancer cells. The results suggest that blocking Per2 or HDAC1 may be a valuable remedy to prevent cancer metastasis.

## Materials and Methods

### Reagents

The reagents for real time RT-PCR, methylation specific PCR and Western blotting were purchased from Invitrogen (Shanghai, China). The antibodies of Period 2, E-cadherin, HDAC1, pHDAC1 were purchased from Bio-Mart (Shanghai, China). The shRNA kits of Per2, HDAC1 and E-cadherin were purchased from Santa Cruz (Shanghai, China). The protein G was purchased from Sigma Aldrich (Shanghai, China). The ChIP kit was purchased from Haoranbio (Shanghai, China).

### Ethic statement

The using human tissue in the present study was approved by the Human Ethic Committee at Hebei Medical University. The procedures were performed in accordance with the guidelines. An informed written consent was obtained from each subject.

### Patients and esophageal cancer tissue collection

Twenty squamous esophageal cancer patients (male = 10; female = 10; age: 33–65 years old) were recruited into the present study, in which 11 cases had lymph node metastasis. The diagnosis of esophageal cancer was carried out by their surgeons and pathologists. All patients did not receive radiotherapy or chemotherapy. The esophageal cancer was surgically removed at our hospital. The esophageal cancer tissue was collected from the operation room of our hospital.

### Isolation of esophageal cancer cells

The esophageal cancer tissue was cut into small pieces (2 × 2 × 2 mm) and incubated in DMEM culture media containing collagenase IV (0.5 mg/ml) for 2 h with mild agitation. The cells were passed through a cell strainer (70 μm). After washing with fresh medium, the cells were centrifuged; the cell pellets were resuspended in medium containing magnetic antibodies of CD3, CD11c, CD14, CD19, NK1.1, CD117 and CD69. The esophageal cancer cells were negatively isolated by the magnetic cell sorting (MACS).

### Cell culture

The cells were cultured in DMEM supplemented with 10% fetal bovine serum (FBS), 100 U/ml penicillin, 0.1 mg/ml streptomycin and 2 mM L-glutamine. The medium was changed in 1 or 2 days. The cell viability was checked with the Trypan blue exclusion assay.

### NCell lines

The human esophageal squamous cell carcinoma cell line EC109 and CP-C cells were purchased from the Cell Bank of Shanghai Institute of Cell Biology (Shanghai, China). The cells were cultured in DMEM. The EC109 cells are a human esophageal squamous carcinoma cell line. CP-C cells are a Barrett’s esophageal cancer cell line.

### Real time quantitative RT-PCR (RT-qPCR)

The total RNA was extracted from the cells with TRIzol reagents. The cDNA was synthesized using a reverse transcription kit following the manufacturer’s instructions. The qPCR was performed on a real time PCR device (MiniOpticon, Bio-Rad) with the SYBR Green Master Mix. The results were calculated by the 2^−∆∆Ct^ method and normalized to the control samples. The primers using in this study are listed in Table 1.

### Western blotting

The total proteins were extracted from the cells; the protein levels were quantified by the Bio-Rad protein assay. SDS-PAGE was performed to fraction the proteins, which were transferred onto a PVDF membrane. The membrane was blocked with 5% skim milk for 30 min at room temperature, incubated with the primary antibodies (0.5 μg/ml) overnight at 4 °C, and followed by incubation with the secondary antibodies (conjugated with peroxidase) for 1 h at room temperature. The blots on the membrane were developed with ECL. The results were recorded by photographing with a KODAK Image Station (4000Pro, KODAK, Shanghai, China). The integrated density of the blots was determined by the software ImageJ, and presented as a percentage of the internal control β-actin.

### Gene silence

The gene silence of the genes of Per2, HDAC1 and E-cadherin was performed by transducing the lentivius carrying Per2 shRNA, or HDAC1 shRNA, or E-cadherin shRNA with commercial reagent kits following the manufacturer’s instructions. The effect of gene silence was determined by Western blotting.

### Overexpression of Per2 in esophageal cancer cells

The whole length of the Per2 gene plasmid was constructed by the GeneScript (Nanjing, China). The Per2 pcDNA plasmids were transfected into the Eca109 cells with the lipofectamine 2000 transfection reagent following the manufacturer’s instructions. The Per2 overexpression in the esophageal cancer cells was assessed by Western blotting.

### HDAC1 reporter assay

The HDAC1 gene luciferase reporter was constructed by the GeneScript (Nanjing, China) and transfected to esophageal cancer cells with or without the Per2 overexpression following the manufactuer’s instructions. The cells were harvested 24 h later and lysed with a lysis buffer. Luciferase assays were performed on an Orion II microplate luminometer (Berthold detection systems, Oak Ridge, TN, Germany) with commercial reagent kits following the manufacturer’s instructions.

### Chromatin immunoprecipitation assay (ChIP)

The esophageal cancer cell E-cadherin promoter locus DNA remolding was analyzed by ChIP with a ChIP reagent kit following the manufacturer’s instructions. Briefly, the esophageal cancer cells were fixed by 1% paraformaldehyde for 15 min and treated with sonication. The cell lysates (input) were precleared by incubating with protein G agarose beads for 4 h at 4 °C. The supernatant was immunoprecipitated with indicated antibodies (2 μg each) and protein G agarose beads. The precipitated protein-DNA complexes were eluted from the agarose beads with an eluting buffer. After cross-linking reversal and proteinase K treatment, DNA samples were precipitated and purified with a DNA purification kit. The DNA was analyzed by qPCR with the primers of E-cadherin promoter region (gctcacgcctgtaatccaac and ccgggttcaagagactctcc). The results were presented as the folds of change against the input.

### *In vitro* migration and invasion assays

esophageal cancer cells with or without Per2 overexpression were seeded in each insert (10^4^ cells) of Transwell (Falcon; 8 μm pore size) and cultured in DMEM. Sixteen hours later, the inserts were fixed with 100% methanol and stained with 5% Giemsa solution. The cells remained on the top side of the supporting filter membrane were removed with a rubber blade; the cells on the reverse side were regarded as migrated cells. The membranes were mounted on glass slides. The cells were counted under a light microscope and photographed at ×200.

### Statistics

The data are presented as mean ± SD. The differences between groups were determined with the Student t test or ANOVA if more than two groups. A p < 0.05 was set as a significant criterion.

## Additional Information

**How to cite this article**: Li, S. *et al.* Modulation of E-cadherin expression promotes migration ability of esophageal cancer cells. *Sci. Rep.* **6**, 21713; doi: 10.1038/srep21713 (2016).

## Supplementary Material

Supplementary Information

## Figures and Tables

**Table 1:** Primers.

Molecules	Forward	Reverse
NFIL3	tcggaggaaacgggaattca	ggcgttttcttctcccagtg
Per1	tgcacctcctttctaccctg	ggcaattcctccatctgcag
Per2	tcctacgttgtggaccagac	cggatgcggcaataaaagga
Bmal1	ccctgggccatctcgattat	tcatccagccccatctttgt
Cry1	cgaggtttccgtgacgaatc	ggcgcagtggaaagatgaat

## References

[b1] Napier K., Scheerer M. & Misra S. Esophageal cancer: A Review of epidemiology, pathogenesis, staging workup and treatment modalities. World J Gastrointest Oncol 6, 112–120 (2014). 24834141 10.4251/wjgo.v6.i5.112 PMC4021327

[b2] Sgourakis G. *et al.* The use of neural networks in identifying risk factors for lymph node metastasis and recommending management of t1b esophageal cancer. Am Surg 78, 195–206 (2012). 22369829

[b3] Shah R., Cassano A. & Neifeld J. Neoadjuvant therapy for esophageal cancer. World J Gastrointest Oncol 6, 403–406 (2014). 25320656 10.4251/wjgo.v6.i10.403 PMC4197431

[b4] Rubenstein J. H. & Chen J. W. Epidemiology of gastroesophageal reflux disease. Gastroenterol Clin North Am 43, 1–14 (2014). 24503355 10.1016/j.gtc.2013.11.006

[b5] Parri M. & Chiarugi P. Rac and Rho GTPases in cancer cell motility control. Cell Commun Signal 8, 23 (2010). 20822528 10.1186/1478-811X-8-23 PMC2941746

[b6] Zheng J. *et al.* Huaier polysaccharides suppresses hepatocarcinoma MHCC97-H cell metastasis via inactivation of EMT and AEG-1 pathway. Int. J. Biol. Macromol. 64, 106–110 (2014). 24321491 10.1016/j.ijbiomac.2013.11.034

[b7] Yamato M. *et al.* E-cadherin and claudin-4 expression has circadian rhythm in adult rat kidney. J Nephrol 23, 102–110 (2010). 20091493

[b8] Okabe T. *et al.* The impact of HIF1alpha on the Per2 circadian rhythm in renal cancer cell lines. PLoS One 9, e109693 (2014). 25333958 10.1371/journal.pone.0109693 PMC4204850

[b9] Haus E. & Smolensky M. Shift work and cancer risk: potential mechanistic roles of circadian disruption, light at night and sleep deprivation. Sleep Med Rev 17, 273–284 (2013). 23137527 10.1016/j.smrv.2012.08.003

[b10] Mazzoccoli G., Vinciguerra M., Papa G. & Piepoli A. Circadian clock circuitry in colorectal cancer. World J. Gastroenterol. 20, 4197–4207 (2014). 24764658 10.3748/wjg.v20.i15.4197 PMC3989956

[b11] Buda A. & Pignatelli M. E-cadherin and the cytoskeletal network in colorectal cancer development and metastasis. Cell Commun. Adhes. 18, 133–143 (2011). 22176698 10.3109/15419061.2011.636465

[b12] Coffey K. *et al.* Characterisation of a Tip60 specific inhibitor, NU9056, in prostate cancer. PLoS ONE 7, e45539 (2012). 23056207 10.1371/journal.pone.0045539 PMC3466219

[b13] Duong H. A., Robles M. S., Knutti D. & Weitz C. J. A molecular mechanism for circadian clock negative feedback. Science 332, 1436–1439 (2011). 21680841 10.1126/science.1196766 PMC3859310

[b14] Hugo H. J. *et al.* Defining the E-cadherin repressor interactome in epithelial-mesenchymal transition: the PMC42 model as a case study. Cells Tissues Organs 193, 23–40 (2011). 21051859 10.1159/000320174

[b15] Becker K. F. *et al.* Analysis of the E-cadherin repressor Snail in primary human cancers. Cells Tissues Organs 185, 204–212 (2007). 17587826 10.1159/000101321

[b16] Peinado H., Olmeda D. & Cano A. Snail, Zeb and bHLH factors in tumour progression: an alliance against the epithelial phenotype? Nat Rev Cancer 7, 415–428 (2007). 17508028 10.1038/nrc2131

[b17] Yun S. J. & Kim W. J. Role of the epithelial-mesenchymal transition in bladder cancer: from prognosis to therapeutic target. Korean J Urol 54, 645–650 (2013). 24175036 10.4111/kju.2013.54.10.645 PMC3806986

[b18] Yun S. & Kim W. Role of the epithelial-mesenchymal transition in bladder cancer: from prognosis to therapeutic target. Korean J Urol 54, 645–650 (2013). 24175036 10.4111/kju.2013.54.10.645 PMC3806986

[b19] Sgourakis G. *et al.* Detection of lymph node metastases in esophageal cancer. Expert Rev Anticancer Ther 11, 601–612 (2011). 21504265 10.1586/era.10.150

[b20] Leloup J. & Goldbeter A. Modeling the circadian clock: from molecular mechanism to physiological disorders. Bioessays 30, 590–600 (2008). 18478538 10.1002/bies.20762

[b21] Evans J. A. & Davidson A. J. Health consequences of circadian disruption in humans and animal models. Prog Mol Biol Transl Sci 119, 283–323 (2013). 23899601 10.1016/B978-0-12-396971-2.00010-5

[b22] Marcheva B. *et al.* Circadian clocks and metabolism. Handb Exp Pharmacol, 217, 127–55 (2013). 23604478 10.1007/978-3-642-25950-0_6 PMC4089089

[b23] Monsees G. M., Kraft P., Hankinson S. E., Hunter D. J. & Schernhammer E. S. Circadian genes and breast cancer susceptibility in rotating shift workers. Int J Cancer 131, 2547–2552 (2012). 22473669 10.1002/ijc.27564 PMC3408553

[b24] Dalvin L. A. & Fautsch M. P. Analysis of Circadian Rhythm Gene Expression With Reference to Diurnal Pattern of Intraocular Pressure in Mice. Invest Ophthalmol Vis Sci 56, 2657–2663 (2015). 25813988 10.1167/iovs.15-16449 PMC4416542

[b25] Moore S. R. *et al.* Robust circadian rhythms in organoid cultures from PERIOD2::LUCIFERASE mouse small intestine. Dis Model Mech 7, 1123–1130 (2014). 24997189 10.1242/dmm.014399 PMC4142732

[b26] Huisman S. A. *et al.* Colorectal liver metastases with a disrupted circadian rhythm phase shift the peripheral clock in liver and kidney. Int J Cancer 136, 1024–1032 (2015). 25045881 10.1002/ijc.29089

[b27] Runkle E. & Mu D. Tight junction proteins: from barrier to tumorigenesis. Cancer Lett. 337, 41–48 (2013). 23743355 10.1016/j.canlet.2013.05.038 PMC3752309

[b28] Liu J. *et al.* Epithelial-to-mesenchymal transition in human esophageal cancer associates with tumor progression and patient’s survival. Int J Clin Exp Pathol 7, 6943–6949 (2014). 25400779 PMC4230146

[b29] Papagerakis S. *et al.* The circadian clock in oral health and diseases. J. Dent. Res. 93, 27–35 (2014). 24065634 10.1177/0022034513505768 PMC3865791

[b30] Zhao H. *et al.* Prognostic relevance of Period1 (Per1) and Period2 (Per2) expression in human gastric cancer. Int J Clin Exp Pathol 7, 619–630 (2014). 24551282 PMC3925906

[b31] Meng F., Sun G., Zhong M., Yu Y. & Brewer M. Inhibition of DNA methyltransferases, histone deacetylases and lysine-specific demethylase-1 suppresses the tumorigenicity of the ovarian cancer ascites cell line SKOV3. Int. J. Oncol. 43, 495–502 (2013). 23709006 10.3892/ijo.2013.1960

[b32] Kou X., Hao T., Meng Z., Zhou Y. & Gan Y. Acetylated Sp1 inhibits PTEN expression through binding to PTEN core promoter and recruitment of HDAC1 and promotes cancer cell migration and invasion. Carcinogenesis 34, 58–67 (2012). 23104175 10.1093/carcin/bgs336

