# Supplementary material for: Modulation of E-cadherin expression promotes migration ability of esophageal cancer cells
Source: Sci Rep. 2016 Feb 22;6:21713. doi: 10.1038/srep21713 (PMC4761978; doi:10.1038/srep21713)
Supplement: Supplementary Information [file srep21713-s1.pdf]

Modulation of E-cadherin expression promotes migration ability of esophageal cancer cells

Running title: Per2 compromises cell-cell junction integrity

Shujun Li[1], Song Cai[1], Changbao Zhai[1], Xuebo Qin[1], Xiaolu Wang[1], Helin Zhang[2]

1, Department of Thoracic Surgery, the Second Affiliated Hospital, Hebei Medical University, Shijiazhuang, 050051, China. 2, Hebei Provincial Thoracic Hospital, Shijiazhuang, 050051, China.

Corresponding author: Dr. Xiaolu Wang, Department of Thoracic Surgery, the Second Affiliated Hospital, Hebei Medical University, Shijiazhuang, 050051, China. Email: xiaolurwang@outlook.com.

Full-length pictures of gel graphs

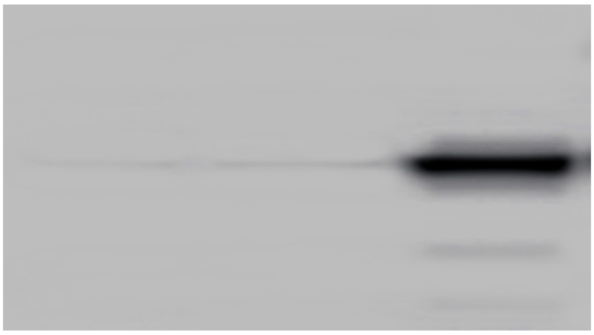

Fig.1C-Per2

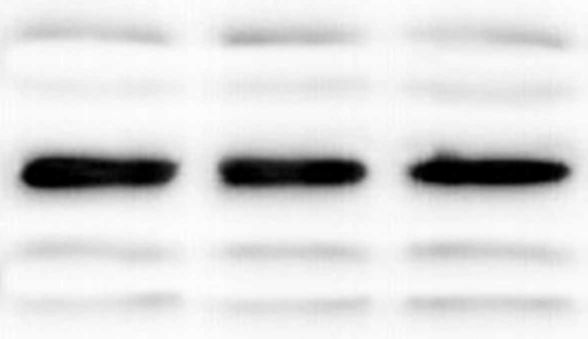

Fig.1C-beta-actin

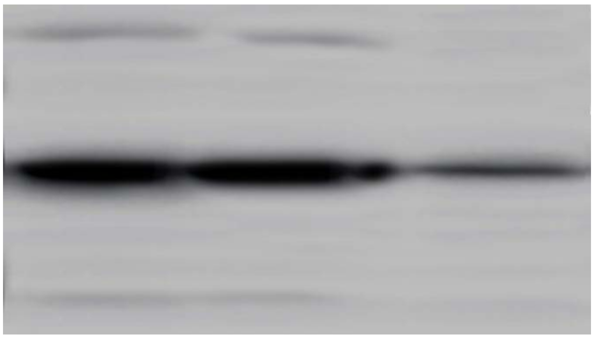

Fig.3D. EC

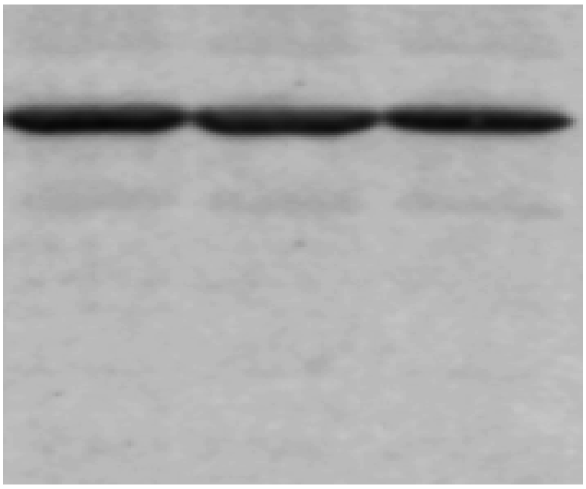

Fig.3D.beta-actin

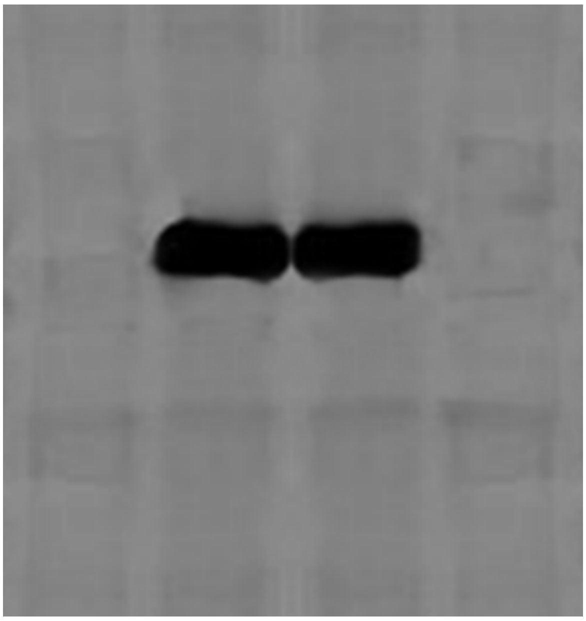

Fig.2A.Per2

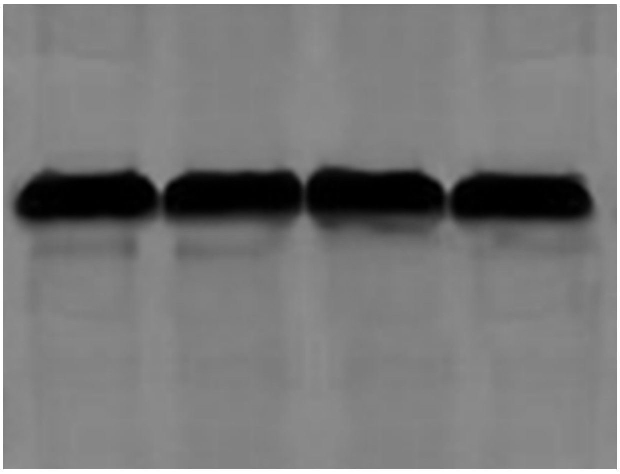

Fig.2A-beta actin

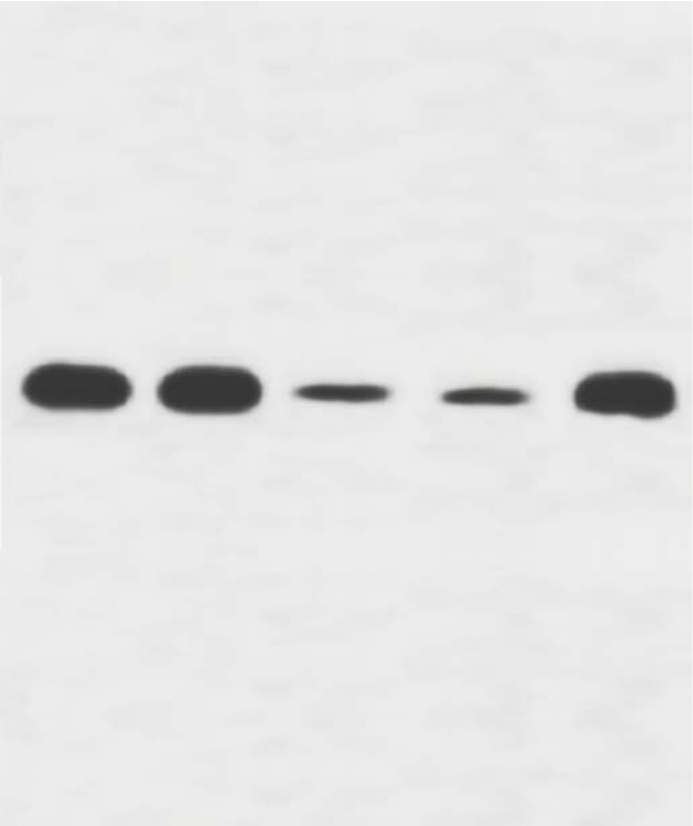

Fig.2C-EC

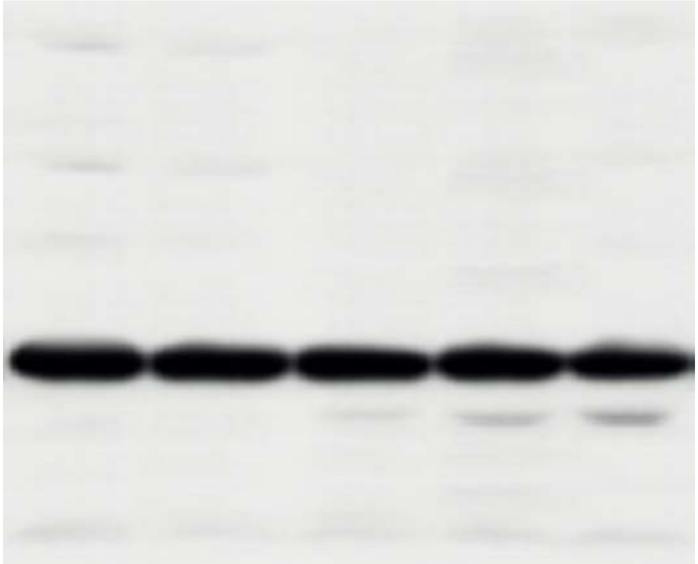

Fig.2C-beta-actin

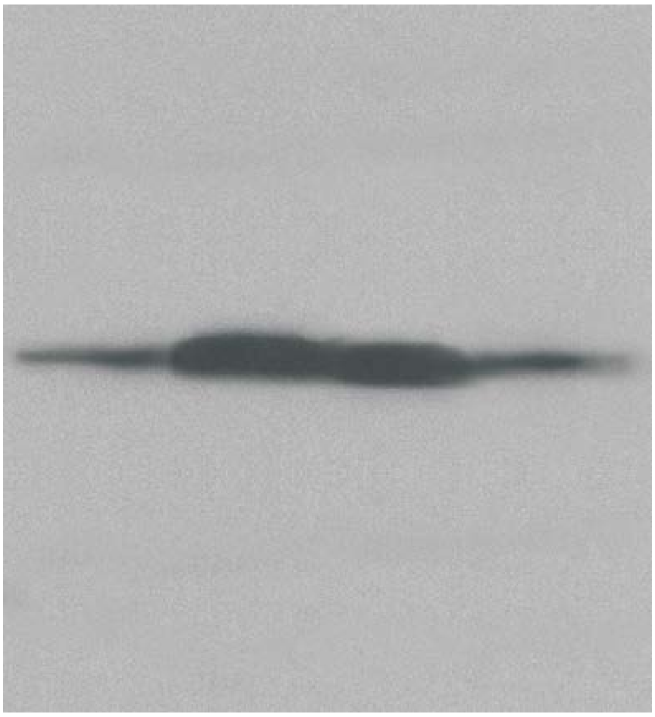

Fig3. pHDAC1

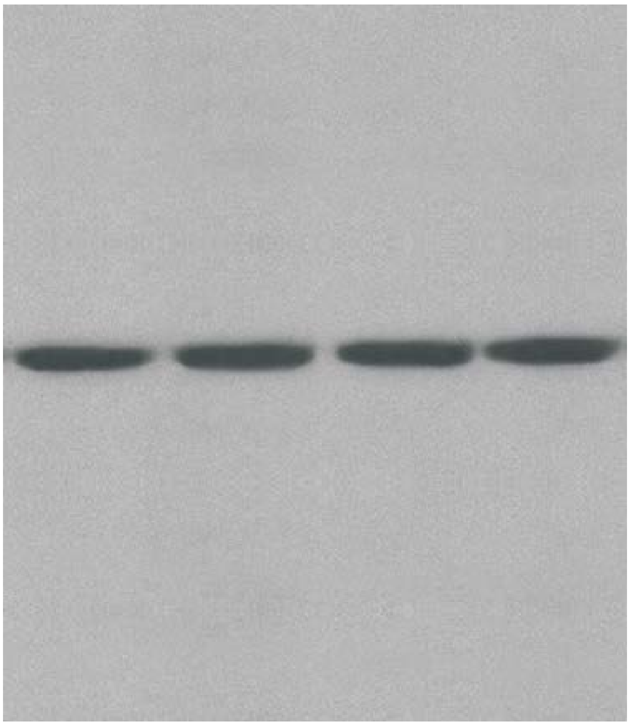

Fig.3. Beta actin

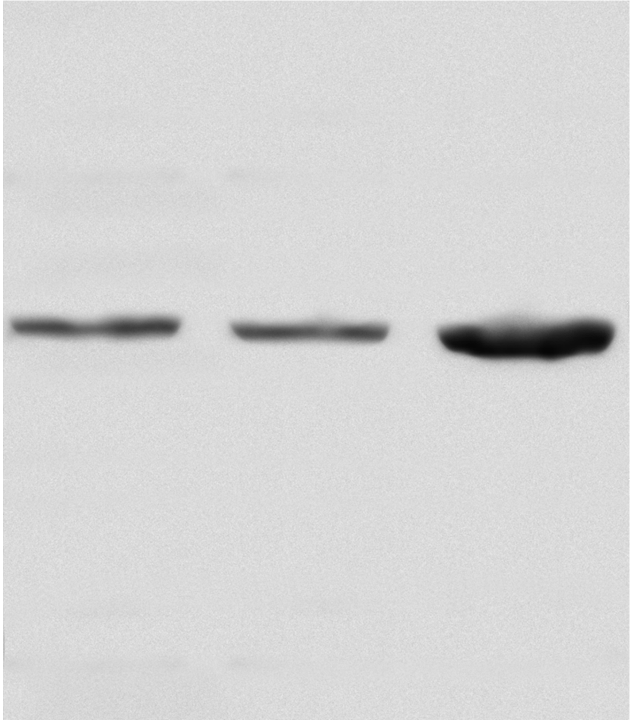

Fig. 3. pHDAC1

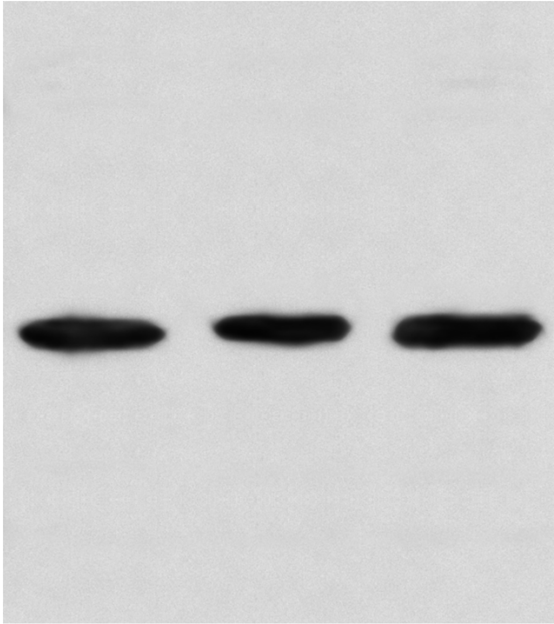

Fig. 3. Beta actin

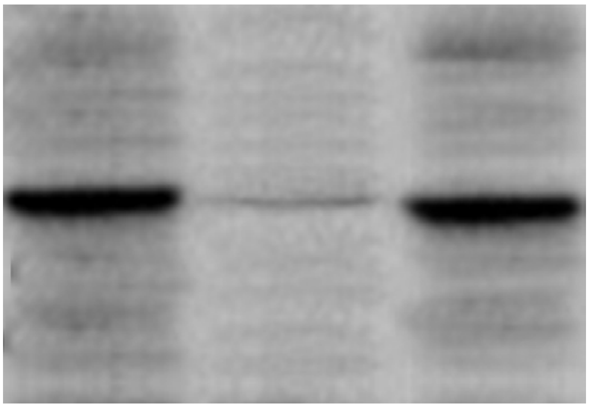

Fig5I-EC

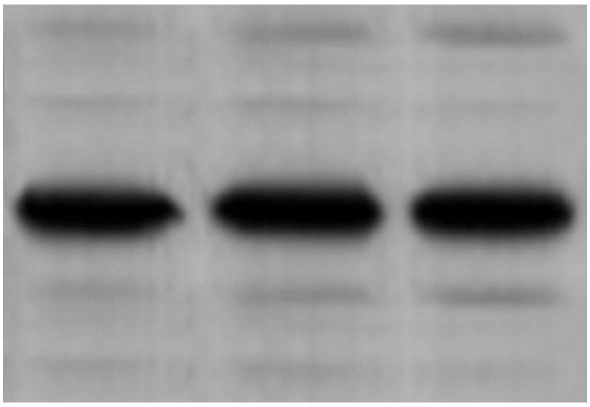

Fig5I-beta-actin
